# Impact of somatostatin interneurons on interactions between barrels in plasticity induced by whisker deprivation

**DOI:** 10.1038/s41598-022-22801-0

**Published:** 2022-10-26

**Authors:** G. Dobrzanski, R. Zakrzewska, M. Kossut, M. Liguz-Lecznar

**Affiliations:** Nencki Institute of Experimental Biology, Polish Academy of Sciences, Pasteur 3, 02-093 Warsaw, Poland

**Keywords:** Sensory processing, Barrel cortex, Whisker system

## Abstract

The activity of inhibitory interneurons has a profound role in shaping cortical plasticity. Somatostatin-expressing interneurons (SOM-INs) are involved in several aspects of experience-dependent cortical rewiring. We addressed the question of the barrel cortex SOM-IN engagement in plasticity formation induced by sensory deprivation in adult mice (2–3 months old). We used a spared vibrissa paradigm, resulting in a massive sensory map reorganization. Using chemogenetic manipulation, the activity of barrel cortex SOM-INs was blocked or activated by continuous clozapine N-oxide (CNO) administration during one-week-long deprivation. To visualize the deprivation-induced plasticity, [^14^C]-2-deoxyglucose mapping of cortical functional representation of the spared whisker was performed at the end of the deprivation. The plasticity was manifested as an extension of cortical activation in response to spared vibrissae stimulation. We found that SOM-IN inhibition in the cortical column of the spared whisker did not influence the areal extent of the cortex activated by the spared whisker. However, blocking the activity of SOM-INs in the deprived column, adjacent to the spared one, decreased the plasticity of the spared whisker representation. SOM-IN activation did not affect plasticity. These data show that SOM-IN activity is part of cortical circuitry that affects interbarrel interactions underlying deprivation-induced plasticity in adult mice.

## Introduction

Since the breakthrough discoveries, proving the existence of massive cortical plasticity caused by denervation in adult animals^1,2^, the traditional view, claiming that such plasticity occurs exclusively around the critical period, where a peak of plastic capabilities of the cortex is observed, has passed into oblivion. Nevertheless, the adult cortex is far less plastic compared to that around the critical period. Thus, a lot of effort is made to uncover the mechanisms governing the plastic changes of the adult cortex. Such knowledge could be valuable when searching for interventions (e.g. pharmacological ones) against detrimental effects of stroke and brain damage or treating developmental conditions, like lifelong amblyopia^3^.

With an essential role in cortical physiology, excitatory neurons comprise up to 85% of all neuronal cells of the neocortex, compared to only about 15% of inhibitory interneurons; however, the link between function and morphological complexity is far more pronounced in the latter group, pointing to a multiple roles of inhibitory interneurons in the functioning of the neocortex^4^. Indeed, by the multi-level impact on the activity of excitatory neurons, neocortical inhibitory interneurons are engaged, often in a causative way in a plethora of brain processes and states, including the sensory deprivation-induced plasticity of the adult cortex^5–8^. In the rodent somatosensory cortex, there are three, non-overlapping neurochemical groups of inhibitory interneurons, and each expresses a characteristic marker: parvalbumin (PV), somatostatin (SOM), or vasoactive intestinal peptide (VIP)^9^. Soma-targeting parvalbumin-expressing interneurons (PV-INs), with a crucial role in maintaining a balance between excitation/inhibition during a developmental critical period, have been the most studied inhibitory cells in mechanisms of sensory deprivation-induced plasticity so far (reviewed in^10^). Along with these studies, PV-IN-related plasticity brake has been reported, removal of which increases an adult cortical plasticity^7^. Nowadays, attention is being shifted into the second largest group on cortical inhibitory interneurons—somatostatin-expressing interneurons (SOM-INs)—and their involvement in neuroplasticity processes is increasingly reported (reviewed in^11^). SOM-INs constitute a heterogeneous group of cells, with different electrophysiology and connectivity patterns, affecting both excitatory neurons and other types of inhibitory interneurons, including PV-INs (reviewed in^12^). Recently, a decreased activity of SOM-INs was hypothesized to orchestrate a closure of the critical period in the visual cortex^13^. Transplantation of precursors of SOM-INs or PV-INs reopened a critical period for ocular dominance plasticity after its closure^14^. SOM-IN activity was proposed in cross-modal plasticity in adult animals^15^; Scheyltjens and colleagues with optogenetic manipulation proved a causal role of V1 SOM-INs in the modulation of sensory integration upon the loss of sensory inputs. Finally, SOM-IN activation and PV-IN deactivation, obtained by overexpression of endogenous positive modulator of nicotinic receptors, restored developmental deprivation-induced plasticity in the adult visual cortex^8^.

A vast majority of results concerning the function of inhibitory interneurons in mechanisms of cortical plasticity are derived from the visual cortex, thus the role of SOM-INs in adult plasticity induced by sensory deprivation in other sensory cortices seems to be less explored. In the current study, we aimed to examine the role of the barrel cortex SOM-IN activity in the cortical plasticity induced by sensory deprivation in adult mice (2–3 months old). We have previously found that plasticity of the barrel cortex after sensory deprivation sparing selected whiskers is strongly manifested in cortical layer 4 (L4)^16–18^, thus in the current study we focused on that layer. With 2-DG and c-fos mapping, it has been already shown that one week of sensory deprivation is sufficient to induce changes in the activation pattern of the adult barrel cortex^17^. Some previous studies from the barrel cortex have indicated stronger synaptic plasticity of inhibitory low threshold-spiking (LTS) cells within deprived barrels compared to non-deprived barrels after sensory loss^19^. Given the fact that LTS cells share many electrophysiological characteristics with SOM-INs (reviewed in^11^), here with a chemogenetic approach, we aimed to compare the role of the barrel cortex SOM-INs of spared and deprived barrels in plasticity formation induced by partial whisker trimming in adult mice. We showed that sensory deprivation-induced plasticity of the adult barrel cortex is decreased after chemogenetic blockade of SOM-IN activity in the cortical column of the deprived whisker adjacent to the spared one during the whole deprivation paradigm (1 week), but inactivation of SOM-INs in the column of the spared whisker did not affect the plastic change.

## Results

**Figure 1:**
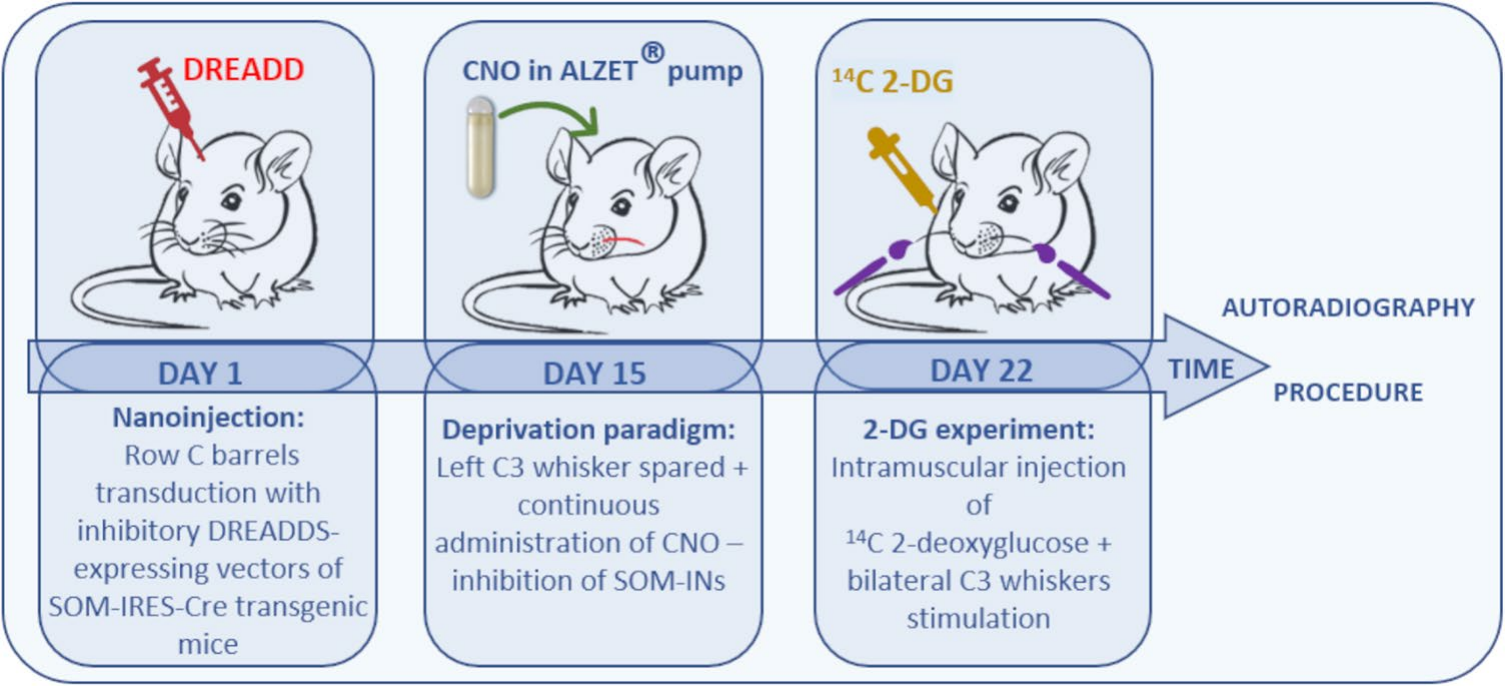
Experimental scheme and timeline. Two weeks after nanoinjection of DREADDs-expressing viral vectors, mice started a one-week-long sensory deprivation paradigm (Day 15), in which all but C3 whisker on the left side of the snout were trimmed (whiskers on the right side of the snout remained untouched) and CNO (1 mg/ml) or sterile saline (0.9%) were administered by micro-osmotic pump during the whole procedure. One day after the end of deprivation (Day 22), 2-deoxyglucose (2-DG) mapping was performed, followed by euthanasia.

In order to examine the role of SOM-IN activity in the cortical plasticity induced by sensory deprivation in adult mice (2–3 months old), we used a paradigm, in which all but one whisker on the one side of the snout were trimmed (the contralateral side being untouched) for one week. To modulate the activity of SOM-INs we used the chemogenetic DREADD technique^20^. With a combination of transgenic mice line (SOM-IRES-Cre) and Cre-dependent, DREADDs-expressing viral vectors nanoinjections under optical imaging of intrinsic signals, we selectively introduced designer receptors and modulated the activity of SOM-INs within a single barrel during the deprivation paradigm. The barrel cortex plasticity following sensory deprivation was investigated with [^14^C]-2-deoxyglucose brain mapping. Details of the study design and experimental timeline are presented in Fig. 1.

### Optical imaging-based nanoinjections

**Figure 2:**
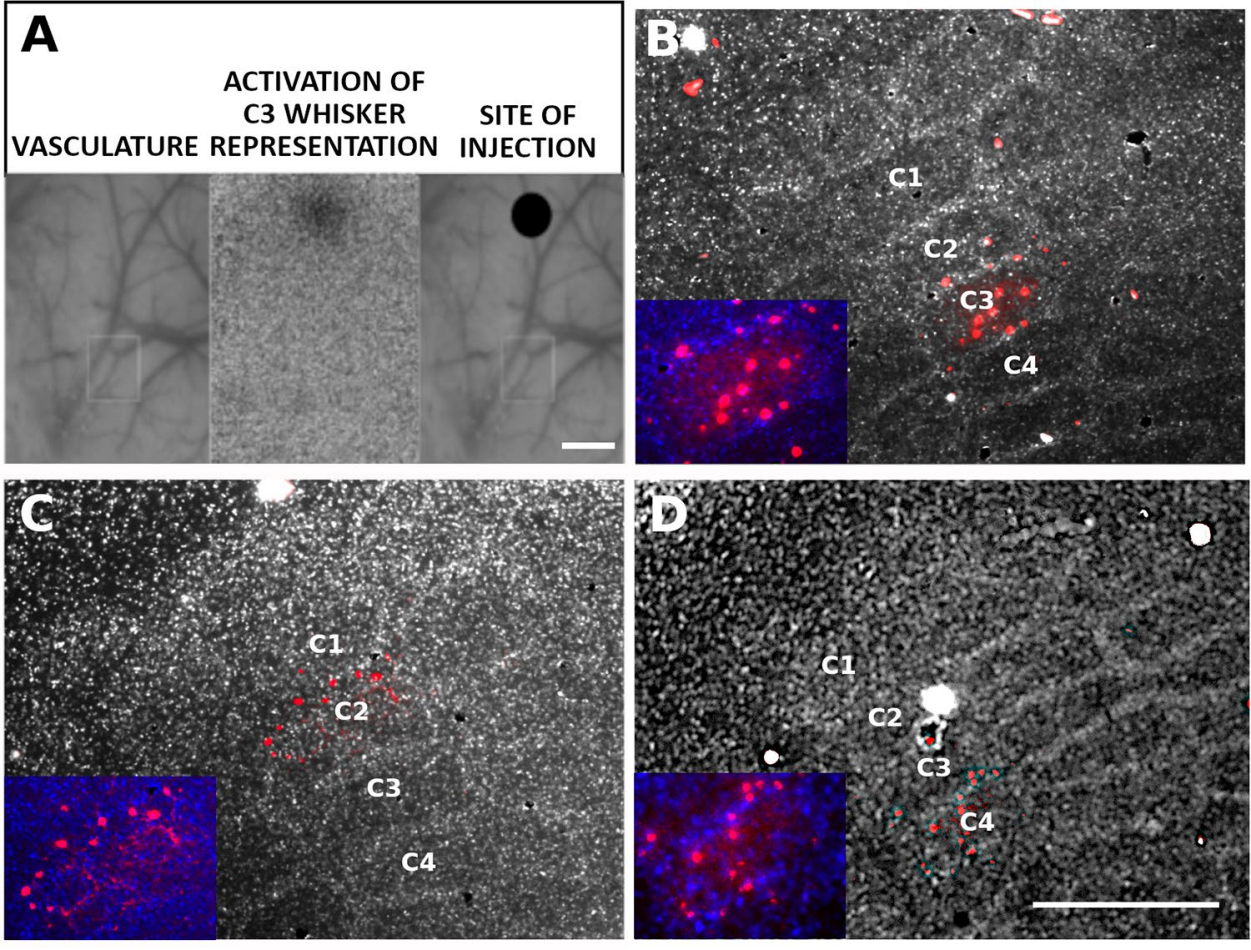
Single barrel nanoinjection under optical imaging of intrinsic signals. (**A**) Images were taken during optical recordings of the activated barrel cortex. Left: an image of the brain vasculature illuminated with green light. Middle: a functional image of the activated C3 representation obtained during C3 whisker stimulation, acquired with orange light illumination. Right: site of injection determined by the superimposition of the two previous images. Scale bar = 500 µm. (**B–D**) Tangential sections of the barrel cortex layer 4 present SOM-IN transduction with DREADDs (red cells) selectively in the representation of C3, C2, or C4 whisker, respectively. Scale bar = 500 µm.

To transduce SOM-INs within a single barrel corresponding to spared or deprived, next to spared whisker, we employed optical imaging-based nanoinjections (Fig. 2A). Since the autoradiography procedure caused the disappearance of fluorescent signal, precluding the detailed examination of layer-specificity of viral transduction, the analysis of the nanoinjection accuracy was performed in every fourth tangential section of the transduced hemisphere, while three fourth of the remaining sections underwent autoradiography procedure. In all tested animals we observed the transduction of virtually all SOM-INs with cell bodies located in the layer 4, and transduction of a few cells of upper layers (as in^21^). Transduction of SOM-INs of deeper layers was very sparse and restricted only to few cells of layer 5a if any were observed. Quantitative analyses showed that L4 SOM-INs constituted about 75% of all transduced cells (74.93 ± 2.93%, mean ± SEM, n = 5). Viral transductions were restricted to the cortical representation of the spared whisker (C3; Fig. 2B) or the representation of deprived, next to spared whisker (C2; Fig. 2C or C4; Fig. 2D). In a separate experiment we have calculated the percentage of inhibitory DREADD-transduced cells among all somatostatin immunopositive cells in layer 4 of the barrel cortex. The results (96 ± 5%, mean ± SD, n = 3) prove a high level of L4 SOM-IN transduction efficiency.

### SOM-IN inhibition within the representation of spared whisker did not change the plasticity

**Figure 3:**
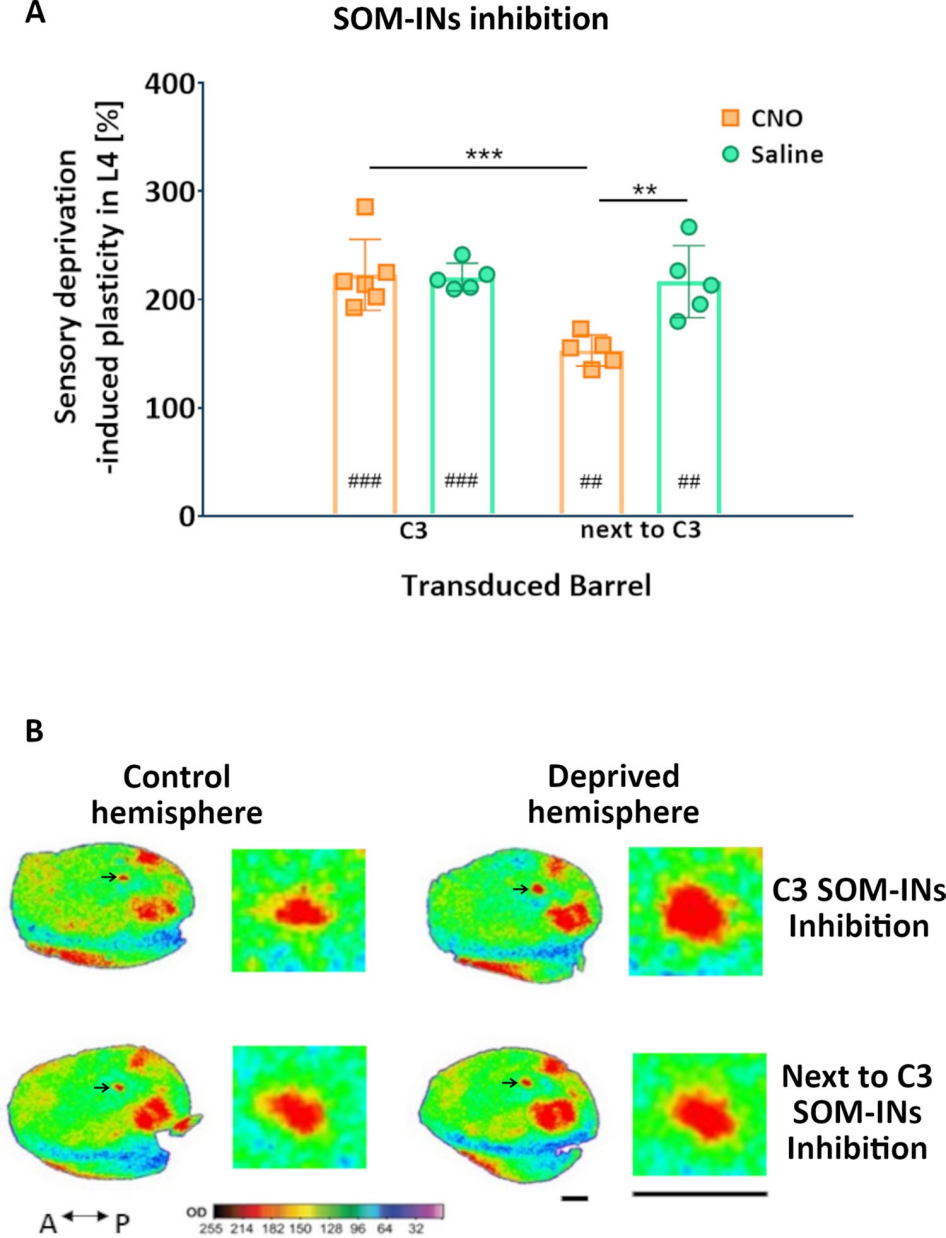
The effect of chemogenetic SOM-IN inhibition on deprivation-induced plasticity. (**A**) Scatter plots presenting the percentage normalized values (mean ± SEM) of the change of sensory deprivation-induced increase in area activated by C3 whisker (analyzed in layer 4) between hemispheres (described as the plasticity, Y-axis) in four groups of animals (X-axis) with SOM-INs transduced with inhibitory DREADDs in C3 barrel and receiving CNO (group no. 1, n = 6) or saline (no. 2, n = 5); SOM-INs transduced with inhibitory DREADDs in next to C3 barrel and receiving CNO (no. 3, n = 5) or saline (no. 4, n = 5). Two-tailed paired t-test showed a significant change in C3 labeling between hemispheres in groups no. 1 (###, p = 0.0003), no. 2 (###, p = 0.0003), no. 3 (##, p = 0.0011) and no. 4 (##, p = 0.0031). Comparison of the plasticity in four groups of animals with two-way ANOVA (1st factor: transduced barrel—C3 or next to C3; 2nd factor: drug treatment—CNO or saline) followed by Bonferroni post-test, revealed a significant change in the plasticity between groups no. 1 vs. no. 3 (***, p < 0.001; F_(1,17)_ = 7.404, p = 0.0145) and no. 3 vs. no. 4 (**, p < 0.01; F_(1,17)_ = 10.74, p = 0.0044). (**B**) Examples of representative, pseudocolored autoradiograms (a pair of control and deprived hemisphere shown in a row) taken from two experimental groups of animals, in which activity of SOM-INs (located either in spared C3 barrel or in deprived barrel next to C3) was blocked during deprivation paradigm. Black arrows indicate C3 labeling, magnified in a square next to the autoradiogram from which it originates. Anterior (A) and posterior (P) sides of autoradiograms are indicated. Scale bar = 1 mm. Pseudocolored autoradiograms and calibration curve were created in Image Lab Software 6.1 (Bio-Rad, https://www.bio-rad.com/).

2-deoxyglucose mapping of the cortical representation of the spared whisker in the control group of animals (saline), revealed that the barrel cortex showed stable labeling of C3 representation in both hemispheres, with the extensive increase of cortical representation of the spared whisker. The mean labeling area of C3 representation in the control hemisphere equaled 98,175 ± 9356 µm^2^ and in the deprived hemisphere 216,203 ± 18,932 µm^2^ (mean ± SEM). The values of C3 labeling taken from both hemispheres passed the normality tests and statistical analysis showed a significant difference in the labeling area of C3 between hemispheres (###, p = 0.0003; two-tailed paired t-test, n = 5). Then, the averaged values from the deprived hemisphere were divided by averaged values from the control hemisphere, multiple by 100. Such a normalized value (here called plasticity) equaled 220.8 ± 5.7% (mean ± SEM) (Fig. 3A). In the control group of animals, in which SOM-INs were transduced with inhibitory DREADDs within C3 representation, but were not activated by CNO, one week of sensory deprivation was sufficient to induce the significant plastic change of the adult barrel cortex. These results also suggest a lack of detrimental effects of viral vectors per se on the metabolic activity of the cortex on 2-DG autoradiograms.

The experimental group of animals, receiving CNO during the deprivation paradigm, revealed a widening of the C3 labeling, corresponding to the spared whisker (Fig. 3B; upper row) (###, p = 0.0003; two-tailed paired t-test, n = 6). The mean labeling area of C3 representation in the control hemisphere equaled 93,223 ± 6628 µm2 and in the deprived hemisphere 202,687 ± 21,735 µm^2^ (mean ± SEM). Deprivation-induced plasticity in this group equaled 222.9 ± 13.4% (mean ± SEM) (Fig. 3A) and was comparable to plasticity observed in the control group of animals (p = 0.8969; two-tailed unpaired t-test), proving that inhibition of the SOM-IN activity in the spared whisker cortical column did not influence the plasticity.

### SOM-IN inhibition in the cortical column of deprived whisker decreased the plasticity

In the next step, we examined the effect of SOM-IN inhibition within the cortical column of deprived whiskers during the deprivation paradigm. Because of the stronger connections along, rather than across barrel rows^22^, we introduced inhibitory DREADDs into the representation of deprived whiskers of the same row, located next to the representation of the spared whisker—into C2 or C4 barrels. Out of ten animals, six were injected into C2 barrel representation and the remaining four into C4, and then a half of C2- as well as C4-targeted animals were randomly assigned to the control group (receiving saline during deprivation procedure, n = 5), and the other half to the experimental group (receiving CNO, n = 5). All other experimental procedures were performed as in previous groups of animals with C3 barrel-targeted nanoinjections.

The control saline group showed wider 2-DG labeling of the spared vibrissa functional representation in the deprived hemisphere (216,513 ± 18,199 µm^2^) than in the control one (97,372 ± 7078 µm^2^) (mean ± SEM, ##, p = 0.0031; two-tailed paired t-test). The average plasticity equaled 216.6 ± 14.9% (mean ± SEM) (Fig. 3A) and it was comparable to that in animals with SOM-IN transduction within C3 [220.8 ± 5.7% (mean ± SEM)]. The mean plasticity of three C2-targeted subgroup of animals was 225.4 ± 21.4%, and of two C4-targeted animals = 203,4 ± 23.4% (mean ± SEM). Since results from both subgroups were similar we decided to pull them together and named the whole group “next to C3”.

The experimental group with C2- or C4-targeted nanoinjection, receiving CNO during deprivation showed a significant decrease in the plastic change within C3 representation (Fig. 3B; lower row), although the plasticity was still present in this group of animals (##, p = 0.0011; two-tailed paired t-test). The mean labeling area of C3 representation in the control hemisphere equaled 105,603 ± 5090 µm^2^ and in the deprived hemisphere 161,156 ± 8088 µm^2^ (mean ± SEM). The mean plasticity of the C3 column in three C2-targeted animals was 157.4 ± 8.4% and was similar to the plasticity found in two C4-targeted animals = 146.6 ± 11.4% (mean ± SEM), so all five values were pulled together. The averaged deprivation-induced plasticity equaled only 153.1 ± 6.4% (mean ± SEM) (Fig. 3A) and it was statistically decreased when compared to the plasticity of the control group (**, p = 0.0044; two-tailed unpaired t-test). To compare the plasticity within the “next to C3” group with plasticity observed in animals, in which activity of SOM-INs was inhibited in the C3 barrel, we employed two-way ANOVA with Bonferroni post-tests, in which one factor was a transduced barrel—“C3” or “next to C3”—and the second factor was a drug treatment—CNO or saline. It demonstrated an effect of transduced barrel (F_(1,17)_ = 10.74, p = 0.0044), drug treatment (F_(1,17)_ = 7.4, p = 0.0145) as well as an interaction between two factors (F_(1,17)_ = 8.45, p = 0.0098). Bonferroni post-tests showed a significant difference in the plasticity of CNO-treated animals, differing in transduced barrel (C3 vs. next to C3) (***, p < 0.001) and in the plasticity of “next to C3” transduced animals, differing in drug treatment during deprivation (CNO vs. saline) (**, p < 0.01) (Fig. 3A). These results point out the relevance of SOM-IN activity in the deprived barrel, but not in the spared one, in cortical plasticity observed after sensory loss.

**Figure 4:**
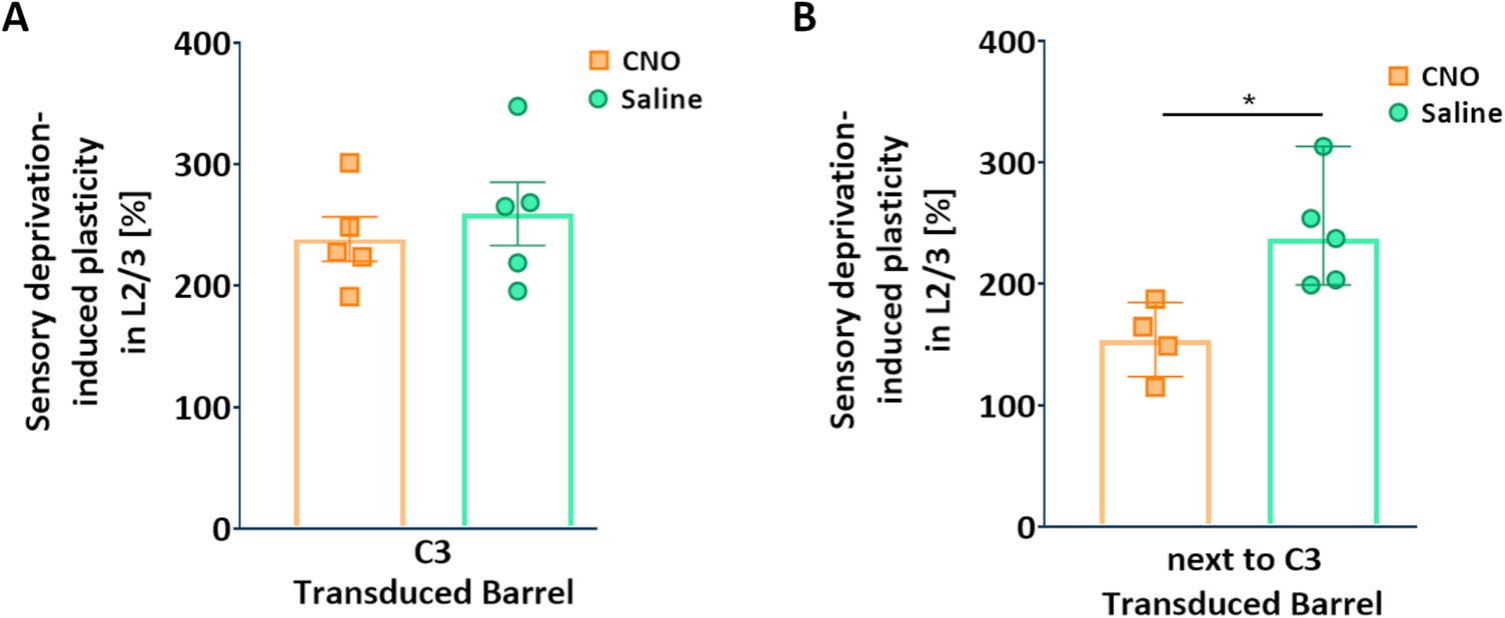
The effect of chemogenetic SOM-IN inhibition on deprivation-induced plasticity in supragranular layers of the barrel cortex. (**A**) Scatter plots presenting the percentage normalized values (mean ± SEM) of the change of sensory deprivation-induced increase in area activated by C3 whisker (analyzed in layer 2/3) between hemispheres (described as the plasticity, Y-axis) in two groups of animals (X-axis) with SOM-INs transduced with inhibitory DREADDs in “C3” barrel and receiving CNO or saline. (**B**) Scatter plots presenting the percentage normalized values (median ± range) of the change of sensory deprivation-induced increase in C3 labeling (analyzed in layer 2/3) between hemispheres (described as the plasticity, Y-axis) in two groups of animals (X-axis) with SOM-INs transduced with inhibitory DREADDs in “next to C3” barrel and receiving CNO or saline. A comparison of the plasticity between the two groups revealed a significant difference in medians (237.4 vs. 157; *, p = 0.0159; Mann–Whitney test).

Interestingly, the decrease in plasticity after SOM-IN inhibition in next to C3 barrel (compared to saline control) was not restricted only to layer 4, but similar observations were done in the upper layers (2 + 3 combined) of the cortical column corresponding to spared C3 whisker, suggesting a profound role of SOM-IN activity in this form of barrel cortex plasticity. In animals with SOM-IN transduction in the representation of spared C3 whisker, the mean plasticity of barrel cortex supragranular layers did not differ between CNO (238.6 ± 18.22%) and saline group (259.4 ± 26.11%) (p = 0.5319; mean ± SEM; two-tailed unpaired t-test, n = 5 per group) (Fig. 4A). However, in the supragranular layers of “next to C3” group, the mean plasticity in the control group equaled 241.4 ± 20.72% (mean ± SEM; n = 5) and the mean plasticity in the experimental group was 154.1 ± 15.16% (mean ± SEM, n = 4). There was a statistically significant difference in medians between the two groups of animals (237.4 vs. 157; *, p = 0.0159; Mann–Whitney test) (Fig. 4B). The reduced number of animals in analyses of layer 2/3 plasticity in “next to C3” group (compared to analyses of layer 4 plasticity) was due to tissue damage during cryostat sectioning.

### SOM-IN activation within the representation of deprived whisker did not influence the plasticity

After demonstrating the decreased plasticity of the barrel cortex as a result of the SOM-IN activity inhibition within the deprived barrels, we examined the effect of the increase of their activity during sensory deprivation. The excitatory DREADDs were introduced into one of the barrels adjacent to C3 barrel (C2 or C4), and during the deprivation paradigm animals were administered with either CNO (experimental group, n = 6) or saline (control group, n = 4).

2-deoxyglucose mapping of the cortical representation of the spared whisker in the control group of animals revealed the increase of cortical representation of the spared whisker. The mean labeling area of C3 representation in the control hemisphere equaled 106,098 ± 2724 µm^2^ and in the deprived hemisphere was 226,271 ± 18,373 µm^2^ (mean ± SEM), yielding the deprivation-induced plasticity of 213.1 ± 15.61% (mean ± SEM).

Autoradiogram analysis taken from the experimental group of animals showed a widening of C3 labeling in layer 4 of the deprived hemisphere, compared to the control one. The mean labeling area of C3 representation in the control hemisphere equaled 106,269 ± 6420 µm^2^ and in the deprived hemisphere was 228,211 ± 17,869 µm^2^ (mean ± SEM). The deprivation-induced plasticity in this group equaled 213.9 ± 7.86% (mean ± SEM).

The between-group comparison of the plasticity (control vs. an experimental group of animals) showed no statistical difference (ns, p = 0.9143; Mann–Whitney test), suggesting a lack of effect of SOM-IN activation within deprived barrels during the deprivation paradigm, on the spared whisker plasticity formation induced by partial whisker trimming.

### CNO administration in DREADD-free animals did not change the plasticity level

To determine the influence of CNO on the plastic change formation, the group of mice (n = 4) underwent all experimental procedures (optical imaging-guided nanoinjections, deprivation procedure, CNO administration, and 2-DG experiment), but during the nanoinjection, sterile saline was administered into the barrel cortex instead of DREADDs-expressing viral vectors.

2-deoxyglucose mapping of the cortical representation of the spared whisker revealed the increase of cortical representation of the spared whisker. The mean labeling area of C3 representation in the control hemisphere was 98,792 ± 2751 µm^2^, and in the deprived hemisphere equaled 236,862 ± 4666 µm^2^ (mean ± SEM). The deprivation-induced plasticity in this group equaled 240.6 ± 10.16% (mean ± SEM). Mann–Whitney test revealed no differences in deprivation-induced plasticity between CNO-only and either hM4Di-transduced (within C3 barrel) CNO-administered (ns, p = 0.2571), or hM3Dq-transduced (next to C3 barrel) CNO-administered group of animals (ns, p = 0.1714). The plasticity comparison between CNO-only DREADD-free and CNO-administered hM4Di-transduced (next to C3 barrel) animals showed a significant difference (*, p = 0.0159, Mann–Whitney test). These analyses suggest that CNO administered alone in DREADD-free animals did not impact the barrel cortex plasticity induced by partial whisker trimming.

## Methods

### Subjects

Transgenic SOM-IRES-Cre mouse line^23^ was acquired from The Jackson Laboratory, USA (stock no. 013044) and bred in the Animal Facility of the Nencki Institute of Experimental Biology, Polish Academy of Sciences (Warsaw, Poland). All 21 mice used in the experiments were male 2- to 3-month-old heterozygotes. Genotyping of SOM-IRES-Cre mice was performed according to protocols provided by The Jackson Laboratory. Before the experiments, all animals were housed in cages with nesting material, four-five per cage, in a temperature- and humidity-controlled room (20–22 °C, 40–50% humidity), on a 12 h light/dark cycle (lights on at 7 a.m.), with ad libitum access to food and water. After the beginning of the experimental procedures, the mice were housed separately under the same conditions. All procedures were consistent with the European Community Council Directive (2010/63/UE) and were approved (approval no. 723/2018) and performed under relevant guidelines and regulations of the First Local Ethics Committee for Animal Experimentation in Warsaw, Poland. The reporting in the manuscript follows the recommendations in the ARRIVE guidelines. All efforts were made to minimize the number of animals used and their suffering.

### DREADD technique

Modulation of SOM-IN activity was achieved by using a chemogenetic DREADD technique^20^, which in many studies was already shown to be an effective approach to change the activity of neocortical SOM-INs in vivo, since it was first demonstrated by Soumier & Sibille (2014)^24^. To introduce DREADDs selectively into SOM-INs we used a combination of transgenic SOM-IRES-Cre mice line, and Cre-dependent, DREADDs-expressing viral vectors. Using immunohistochemistry and whole-cell patch-clamp recordings, we have previously demonstrated the effectiveness of the DREADD technique in modulating the activity of barrel cortex SOM-INs^21^.

### Viral nanoinjections under optical imaging of intrinsic signals

We used optical imaging of intrinsic signal technique^25^ to map a single cortical column corresponding to the selected whisker. Optical imaging of intrinsic signals has been shown to be effective in the guidance of microinjections and electrode insertion into a single cortical column of mice barrel cortex^26^. During optical imaging we stimulated the row C whisker and recorded its cortical activation, localizing its cortical representation. The experimental procedure of the optical recordings of the activated cortex has been described in detail in^21^. After completion of the procedure, the image of the pial surface with blood vessels and the image of row C whisker activation were superimposed on each other, providing the visualization necessary for targeted viral injection. Then, mice were injected with Cre-dependent serotype 2/2 adeno-associated viruses expressing inhibitory (hM4Di) or excitatory (hM3Dq) DREADDs (4.9 × 10^12^ vg/ml of pssAAV-2/2-hSyn1-dlox-hM4D(Gi)_mCherry(rev)-dlox-WPRE-hGHp(A) and 2.8 × 10^12^ vg/ml of pssAAV-2/2-hSyn1-dlox-hM3D(Gq)_mCherry(rev)-dlox-WPRE-hGHp(A), respectively). Vectors were provided by the Viral Vector Facility, University of Zurich.

Selected row C barrel of the right hemisphere was injected with DREADDs-expressing viral vectors (35–40 nl/barrel) using a nanoliter injector (Nanoliter 2010, WPI) ended with a glass capillary backfilled with paraffin oil (catalog no. 76235, Sigma-Aldrich), one injection per barrel. Under the isoflurane (Aerrane, Baxter) anesthesia (3% induction, ~ 2% maintenance) and analgesia [tolfenamic acid (4 mg/kg)], the skull above the selected barrel was thinned with a dental drill, and a small fragment of the skull was lifted to produce an entrance for the injection capillary. Cortical injections were performed perpendicularly to the surface of the brain at a depth of 330 µm from the brain surface, with a flow rate set at 4 nl/min. Body temperature was maintained at 37 °C (Harvard Apparatus, Cambridge, UK), and the breathing rate was monitored (Datex Capnomac Ultima, Finland). After the procedure, the skin was sutured with an absorbable suture (Dafilon, Braun, Germany), and the mice were subcutaneously injected with enrofloxacin (5 mg/kg) and tolfenamic acid (4 mg/kg). These drugs were administered for three consecutive days. After nanoinjection surgery, the mice were housed separately to avoid scratching the wound and plucking out the whiskers by cagemates.

For analysis of the viral injections, every fourth tangential section of the transduced hemisphere was mounted on microscope glass using VECTASHIELD® Antifade Mounting Medium with DAPI (catalog no. H-1200, Vector Laboratories) and red fluorescent protein mCherry (fused to DREADDs) signal was analyzed using fluorescence microscopy. Analysis of layer-specificity of viral transduction was performed in all tested mice.

### Deprivation protocol and DREADD activation

After the nanoinjection procedure, the mice started a two-week-long habituation to a restraining holder (10 min per day), which allowed the mice to be kept in one place and gave easy access to whiskers, to be stimulated during the 2-deoxyglucose procedure. Two weeks of habituation allowed for effective DREADD expression as well. After habituation, the mice were subcutaneously implanted with Alzet® Micro-Osmotic Pumps (model 1007D), which allowed for a continuous drug administration during the whole period of deprivation paradigm. The mice were placed in a plexiglass box and initially anesthetized by inhalation of 3% isoflurane (Aerrane, Baxter). During the implantation surgery, ~ 1.5% isoflurane was provided. Body temperature was maintained at 37 °C (Harvard Apparatus, Cambridge, UK), and the breathing rate was monitored (Datex Capnomac Ultima, Finland). Before implantation, the mice were subcutaneously injected with tolfenamic acid (4 mg/kg), serving as an analgesic. Then, the about 10 mm long skin cut was made on the back of the mice, the pump was inserted and the cut was sutured with an absorbable suture (Dafilon, Braun, Germany), and a warm (37 °C) sterile 0.9% saline was subcutaneously injected around the pump. Before the implantation, pumps were filled with fresh CNO (dissolved in sterile saline to a concentration of 1 mg/ml; catalog no. BML-NS105, Enzo Life Sciences) or sterile 0.9% saline and primed in sterile 0.9% saline for 4 h in 37 °C to begin operating. This CNO concentration was shown to be effective in changing cell activity^27^. After implantation, all whiskers but C3 on the left side of the snout were trimmed. During the one-week-long deprivation paradigm, the trimmed whiskers were checked every two days and were retrimmed, if regrew.

### The 2-deoxyglucose procedure (2-DG)

To visualize a deprivation-induced plastic change of the barrel cortex, [^14^C]-2-deoxyglucose autoradiography was used, a method that allows examining the brain metabolic activity in vivo^28^ and it is widely used to study brain plasticity^18,29,30^.

One week after the beginning of the deprivation, the mice were placed in the restraining holder, and all whiskers except C3 on both sides of the snout were trimmed. Immediately after whisker trimming, the mice were intramuscularly injected with 0.175 ml of [^14^C]-2-deoxyglucose (American Radiolabeled Chemicals, Inc., specific activity 53 mCi/mmol), and both C3 whiskers were stimulated for 30 min, with a frequency of about 2 Hz. Next, the mice were anesthetized with a lethal dose of barbiturate (Vetbutal, Biowet, Poland) and briefly perfused with 4% paraformaldehyde solution in PBS (pH 7.4, Santa Cruz Biotechnology, catalog no. sc-281692). Their brains were removed, and the hemispheres were separated and flattened. The hemispheres were then cut into 30 µm thick serial sections tangential to the barrel cortex on a cryostat (− 20 °C), these were dried on a hot plate and placed, together with [^14^C] radioactive standards, against Kodak X-ray mammography film for one week.

Autoradiograms were scanned with a calibrated densitometer (GS-900™, Bio-Rad), and the scans were pseudocolored using Image Lab™ Software and then analyzed in ImageJ software (1.50i; Wayne Rasband, National Institutes of Health, USA). A calibration curve was created based on the absolute gray levels of the [^14^C] standards. The signals on all analyzed autoradiograms were within the linear range of this curve. The criterion for labeling was that the intensity of 2-DG uptake was at least 15% higher than in the surrounding cortex, as described in Siucinska and Kossut^29^. Labeling of C3 representation fulfilling this criterion was thresholded, and the area of the labeling covering thresholded pixels was measured in µm^2^. The identification of barrels was performed on counterstained with Cresyl Violet sections (Nissl staining), from which the autoradiograms were obtained. The area of the C3 representation was measured in four sections of layer 4, and these four values were averaged for each hemisphere. Averaged values from the deprived hemisphere were divided by averaged values from the control hemisphere, multiple by 100, and such normalized values were presented in figures as a percentage change of sensory deprivation-induced increase in labeling between hemispheres (here defined as the plasticity). Measurements of C3 labeling in layer 2/3 of the barrel cortex were done in tangential sections cut prior to those of layer 4, and the analysis of the plasticity was performed as stated above for layer 4.

### Statistical analyses

Statistical analyses were performed using GraphPad Prism5 software (Inc.). The results are presented as the mean ± SEM, and p < 0.05 was considered statistically significant. A normality test (Kolmogorov–Smirnov test with Dallal-Wilkinson-Lilliefor corrected P-value) was performed to assess the normal distribution of data sets. The comparison of C3 labeling between hemispheres within one group was performed with a two-tailed paired t-test (the symbol # was used to represent a level of significance). A comparison of the plasticity between four groups of animals was performed using two-way ANOVA with Bonferroni post-tests and between two groups with a two-tailed unpaired t-test (the symbol * was used to represent a level of significance). If data did not follow a normal distribution, nonparametric equivalents were used.

## Discussion

We found that in the barrel cortex of adult mice, deprivation-induced plasticity can be shaped by the neighboring barrel’s influence. The outcome of the one-week-long single-whisker experience was different when SOM-IN inhibition was attenuated in the spared whisker representation than in the neighboring, deprived one. Chemogenetic inhibition of SOM-INs in the spared C3 barrel during deprivation did not affect the plastic change of the C3 column. However, SOM-IN inhibition in neighboring barrels (C2 and C4) did, decreasing it by about 30%. On the contrary, activation of SOM-INs in these barrels during deprivation had no effect on the plastic change. Although the SOM-INs were manipulated mostly in L4, the effect extended to supragranular layers.

It needs to be emphasized that the sensory deprivation in the whisker-to-barrel system comes in many forms and the outcome may critically depend on the methodology used. Here we used unilateral trimming, which decreases the sensory input to the contralateral cortical hemisphere, sparing the ipsilateral sensory input. This may cause behavioral compensation and overuse of the spared side, leading to increased activities on the spared side^31^ which may influence the deprived side most probably through corpus callosum connections^32^. We have used the single-whisker experience protocol, in which the spared whisker representation was deprived of competition coming from adjacent whiskers, which in the chessboard paradigm (trimming every second whisker) is postulated to increase the difference in sensory experience of the principal and adjacent whiskers, forcing spared whisker to innervate the deprived barrels^33^. The single-whisker experience was shown to induce the expansion of the spared corresponding barrel, widening its receptive field^34,35^, as well as enhancing the magnitude of neuronal responses to the deflection of the spared whisker^36^.

Based on the research on the developmental plasticity when the critical period for experience-dependent synaptic plasticity is longer in L2/3 than in L4^37^, electrophysiological data suggested cortical L2/3 as the main site for sensory loss-induced plasticity in adult animals^34,38–41^. However, some other studies prove the ability of L4 neurons to undergo plastic changes in adulthood, by pointing out the changes in neuronal responses of L4 neurons^42^, the increase in strength of postsynaptic currents, and the number of synapses at L4 stellate cells^43^. Interestingly, after the sensory loss in the adult barrel cortex, massive structural plasticity of thalamocortical axons^44,45^, and changes in cortical spine density^46^ are observed, further demonstrating the L4 ability for plastic changes. Oberlaender and colleagues^44^ showed that whisker trimming caused a shrinking of the thalamocortical axons innervating the deprived column, hence a decrease in synapses from the thalamus at L4 neurons. They also found that axonal shrinking in the deprived barrel affects responses of layer 4 neurons to sensory stimulation by increasing the level of synchrony among L4 cells, but not the number of action potentials or spontaneous firing rates (which is in line with electrophysiological data from barrel cortex L4 of adult animals). They concluded that if the synchrony of neuronal population influences downstream targets^47^, the observed changes in L4 neuronal synchrony may affect the L4-L2/3 circuit^48^ and eventually the L2/3 synaptic plasticity. Since L4 SOM-INs are important components of L4 circuitry and SOM-INs are known to modulate neuronal synchrony^49–51^, we propose that in our experiments chemogenetic shutting off SOM-IN activity during deprivation could interfere with deprivation-induced L4 synchrony, and attenuate the processes leading to the expansion of spared C3 representation in L4, and eventually in L2/3, visualized with 2-DG.

How the activity from deprived barrel whiskers can influence the spared one? The inhibition in the barrel cortex is executed by intracolumnar, lateral (i.e. cross-columnar), and translaminar inhibition, which has been implicated in feedback as well as feed-forward inhibition^52^. The cross-columnar processing is important for shaping multi-whisker convergence^53–56^, which plays a leading role in defining excitatory and inhibitory parts of receptive fields^57^. Although the horizontal projection system originates mainly from supra- and infragranular layers, a few axonal projections from L4 neurons to adjacent barrel columns also exist^58,59^. A small fraction of L4 spiny neuron axons projects into adjacent barrels, providing interbarrel signaling; some L4 star pyramids exhibit long-range projections over several barrel-columns or rows, both in L4 and in infragranular layers^59^. They show a preference to run along rows rather than arcs of whiskers^60^. Cross-columnar projections are functionally weak in naive animals however, they can be strengthened in experiments forcing unequal use of whiskers^61^. There are two scenarios proposed to explain this phenomenon: first, that branches of thalamic afferents innervating their principal barrels grew into the neighboring deprived barrel^44^, or the other one, that the already existing non-principal whisker input, which normally would be masked by the principal input, is potentiated during the deprivation^56^.

In our study, changes of the inhibitory tone within the deprived whisker barrel during single-whisker experience affected functional plasticity of the spared-whisker representation, while inhibiting SOM-INs in the spared whisker barrel had no effect. One explanation of this phenomenon, consistent with intracolumnar plasticity, would be that response changes of L4 SOM-INs induced by sensory deprivation are manifested exclusively in the representation of deprived but not spared whiskers^19^. This was observed by Li and colleagues^19^ who found an increased amplitude and speedup of the decay rates of spontaneous inhibitory postsynaptic currents in L4 LTS cells of deprived, but not undeprived control barrels after prolonged whisker trimming. Such differences could be an aftermath of axonal plasticity happening in the deprived column^44^. Drew and Feldman^62^ using single-unit electrophysiological recordings observed the weakening of responses to deprived whiskers in L2/3 and L4 as well as isotropic contraction of deprived whisker representations during one-row deprivation. However, other deprivation-induced changes are not restricted to the deprived whisker representation but are rather bidirectional. Jacob et al.^63^ have shown that after ten days of whisker-row deprivation, sensory information from spared whiskers was increased and advanced in L4 and L5 regular spiking neurons. Therefore, it seems that while the plasticity observed in the deprived whisker representations is forced by the intracolumnar mechanisms, the plasticity in spared whisker representation is supported by cross-columnar interactions.

In the Oberlaender and colleagues study, the changed L4 synchrony was proposed to be an aftermath of deprivation-induced morphological changes^44^. The morphological neuronal plasticity was observed in both excitatory and inhibitory neurons in paradigms involving whisker stimulation^64^ and whisker trimming^65^. Sensory loss-induced axonal growth in the barrel cortex L2/3 and L4 has been shown to occur in parallel with functional plasticity studied with 2-DG, in which functional representation of spared whiskers invaded neighboring deprived ones^66^. Marik and colleagues^6^ found an axonal growth of excitatory neurons from the representation of spared whisker towards deprived barrels in the L2/3, while axons of inhibitory interneurons within the representation of the deprived whiskers sprouted long-range projections in the direction of spared barrels, and this process was faster compared to changes of excitatory neurons. The immunohistochemistry experiments revealed that interneurons engaged in the deprivation-induced morphological changes were positive for either calbindin, calretinin or parvalbumin^6^. Since the vast majority of transduced SOM-INs in our experiments were localized in L4, and these cells can target PV-INs^67^, we hypothesize that observed results may stem, at least partially, from the changed PV-IN activity. Among cortical inhibitory interneurons, PV-INs, being preferentially targeted by thalamocortical inputs and having unique electrophysiological characteristics, enable fast and powerful control of excitatory neuron activity^68^. Moreover, since in the somatosensory cortex SOM-INs prefer targeting PV-IN dendrites rather, than PV-IN cell bodies^69^, one of the functions of L4 SOM-INs may be a modulation and synaptic integration of thalamic inputs onto PV-INs. In this manner, they may consolidate inputs reaching L4 and adjust local activity both under physiological conditions and after sensory loss. It was shown that in the barrel cortex, intrinsic properties and other electrophysiological features of L4 PV-INs, are altered after whisker removal^70–72^ and since the axonal plasticity is activity-dependent^73^, we hypothesize that shutting off the L4 SOM-IN activity during whisker deprivation could disrupt PV-IN capability for the deprivation-induced L4 morphological changes, that would eventually be manifested in changes, observed with 2-DG. Assuming that similarly to L2/3, L4 inhibitory interneurons may also sprout long-range projections from deprived to the spared whisker representation (Marik and colleagues^6^), and L4 SOM-INs preferentially target PV-INs rather than excitatory neurons, their activity may be more important in the deprived rather than the spared cortex, shaping the surround inhibition of the spared column.

We cannot rule out the possibility that our results can be partially explained by chemogenetic inhibition of L2/3 SOM-INs Martinotti-type cells, which, next to L4 SOM-INs, were sometimes transduced with DREADDs-expressing viral vectors during L4-targeted nanoinjections. In deprived columns, there is a global decrease in whisker stimulation-evoked neuronal response in L2/3, visualized with calcium imaging^74^, which is in line with electrophysiological reports^38^. Since Martinotti-type SOM-INs control the activity of L2/3 pyramidal excitatory cells, their blockade during whisker trimming could decrease the sensory loss-induced suppression of L2/3 neurons and interfere with plastic change formation. Such a scenario, however, seems less plausible, since it is known that one of the main functions of Martinotti-type SOM-INs is providing feedback inhibition in response to states of local increased activity. If the response of deprived L2/3 neurons is already weakened^74^, no feedback inhibition is needed, and thus it is not likely that blocking L2/3 SOM-INs of deprived columns could affect the observed results.

Observing the positive effect of SOM-IN inhibition in barrels neighboring to spared whisker, we implemented a paradigm of chemogenetic SOM-IN activation in the same barrels during deprivation, which did not affect the plastic change. This result is similar to our observation concerning the effect of SOM-IN activity on learning-induced plasticity^21^.

Here, we report that selective chemogenetic blockade of SOM-INs in the deprived barrels during single-whisker experience decreases the representational plasticity of the spared vibrissal column, visualized with 2-DG. To our knowledge, this is the only report of the involvement of genetically defined somatostatin-expressing interneurons in the mechanisms of adult sensory loss-induced plasticity of the barrel cortex. Since the viral nanoinjections were targeted into L4, the vast majority of all transduced SOM-INs were restricted to this layer, hence to a large extent they mediate the observed results. L4 SOM-INs are important components of barrel cortex L4 circuitry, and we hypothesize that in face of sensory loss their activity may be relevant in maintaining the deprivation-induced L4 synchrony and morphological changes of the deprived barrel, which can eventually lead to expansion of the spared whisker representation in single-whisker experience paradigm.

Although more complex further research is needed to establish the mechanistic role of SOM-IN involvement in the process of deprivation-induced plasticity, here we provide evidence that they are a part of cortical circuitry which shapes the neuroplastic processes in the adult barrel cortex.

## Data Availability

The datasets used and/or analyzed during the current study are available from the corresponding author on reasonable request.
